# Exploring adolescents' and stakeholders' perceptions of online and school‐based mental health provision

**DOI:** 10.1002/jcv2.12288

**Published:** 2024-11-27

**Authors:** Beth T. Bell, Laura Fox, Louisa Salhi, Daniel Fitton

**Affiliations:** ^1^ Psychology in Education Research Centre Department of Education University of York York UK; ^2^ Kooth Digital Health, London United Kingdom and School of Psychology University of Kent Canterbury UK; ^3^ School of Computing and Communication Lancaster University Lancaster UK

**Keywords:** adolescence, help‐seeking, mental health, online support, school, social media, support

## Abstract

**Background:**

Adolescents' engagement with online mental health support (e.g., apps, social media) may affect their engagement with traditional support, including in schools. However, research has typically considered engagement with online‐ and school‐based mental health support separately meaning the mesosystemic interaction between the two is overlooked. Using co‐produced exploratory qualitative methods, the present study explored adolescents' and adult stakeholders' perceptions of how and why adolescents engage with school‐based and online mental health support, the interaction between these two modalities, and the associated risks and opportunities.

**Methods:**

A youth advisory board (*n* = 4; Age = 18–25) co‐created data collection methods, ethics procedures, and data analysis. For the main phase of data collection, 27 adolescents (Age *M* = 15.42, *SD* = 1.58, *Range* = 12–18, *Girls* = 73.1%, White = 84.6%) with mental health difficulties who had engaged with online support while at school were recruited from across England. Participants chose to participate in an interview (*n* = 10) or focus group (*n* = 17, 5 groups). In addition, interviews were conducted with 12 adult stakeholders who worked in fields related youth mental health.

**Results:**

Data was analysed using template analysis resulting in four themes; (a) *Support is abundant, but accessing what you need when you need it is tough*, (b) *Human connection is vital and can be achieved in diverse ways*, (c) *Striving for autonomy and control*, (d) *Navigating credibility and trust across contexts*.

**Conclusions:**

Different types of support met adolescents' needs in different ways, and each offered relative strengths and weaknesses. Findings highlight how adolescents value autonomy and choice when engaging with support, using multiple different sources of support in complementary and self‐directed ways to meet their needs. Several challenges were identified across settings, which could be overcome through increased collaboration. This improved collaboration has potential to improve the quality of support available to adolescents.

## Graphical abstract

Adolescents' engagement with online mental health support may affect their engagement with traditional support in schools. Using coproduced qualitative methods, this study examined the interaction between school‐based and online support from a range of perspectives. Increased collaboration between schools and online services has potential to improve support for adolescents.

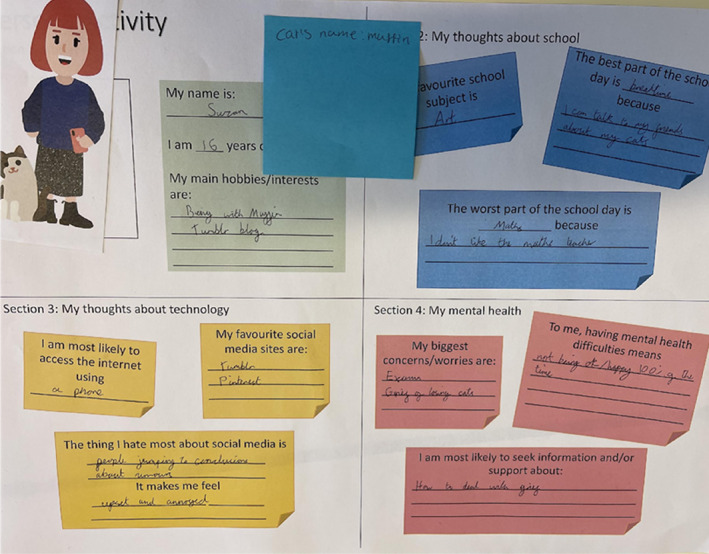

**Key points**

**What's known?**

- Adolescents experiencing mental health difficulties increasingly seek out information and support online.
- Seeking help online can influence adolescents' engagement with traditional services, including at school, but little research has examined this.

**What's new?**

- Adolescents use online and school‐based mental health support in combination with one another in complementary and self‐directed ways to meet their needs.
- Despite the diversity of mental health support available to adolescents, accessing timely, relevant and credible support was a challenge.
- Several challenges were identified across settings, which could be overcome through increased collaboration between schools and online services.

**What's relevant?**

- Online mental health providers and schools should explore ways of collaborating to create complimentary and holistic support for adolescents
- Developing an enhanced mental health literacy curriculum for schools that encompasses the strengths and weaknesses of online support may be useful.

## EXPLORING ADOLESCENTS AND STAKEHOLDERS' PERCEPTIONS OF THE INTERACTION BETWEEN ONLINE AND SCHOOL‐BASED MENTAL HEALTH PROVISION

Youth mental health difficulties are a pressing globe issue. Around 13% of adolescents aged 10–19 live with a diagnosed mental disorder (Unicef, 2023), and in the UK, up to one in six adolescents have a probable mental disorder (NHS Digital, 2021). Furthermore, the number of UK‐based young people reporting mental health difficulties has increased by 50% in the last 3 years (The Good Childhood Report, 2022). Mental health difficulties can have a significant impact on adolescent functioning, contributing to negative social, economic and health outcomes (Clayborne et al., 2019). Many adolescent mental health problems persist into adulthood, but some do not (Girela‐Serrano et al., 2023; Patton et al., 2014). Studies show that adolescents who engage in support are more likely to experience a reduction in negative mental health symptoms than those who don't (Neufeld et al., 2017). As such, providing appropriate support to adolescents with mental health difficulties is vitally important.

Schools play a crucial role in combatting youth mental health difficulties (Hoover & Bostic, 2021), providing formal and informal support to young people through the provision of education, prevention efforts, early interventions, and referrals to specialist services (Department for Education, 2018). As many as two thirds of adolescents with mental health difficulties will discuss their problems with teachers (Newlove‐Delgado et al., 2015). However, providing comprehensive mental health support in schools is a challenge. School staff often lack specialist mental health training and lack confidence in their ability to support students (O’Farrell et al., 2023; Samnøy et al., 2022). In a survey of over 1000 schools across 10 European countries, further barriers to support included limited staff capacity (e.g., time), limited funding, and poor specialist support access (Patalay et al., 2016). Adolescents also experience additional social barriers to support (e.g., stigma, bullying) and have additional concerns surrounding confidentiality and privacy related to their disclosures (Pohlman et al., 2002). Thus, there are multiple challenges in play which may mean that school mental health support does not always meet adolescents' needs.

Fortunately, schools represent just one cog in the mental health support ecosystem, and other sources of support are available ‐ including online. Adolescents increasingly use digital technologies and online services for mental health support (Pretorius et al., 2019a, 2019b); Pretorius, Chambers, & Coyle, 2019 trend that was intensified during the covid‐19 pandemic (Yonemoto & Kawashima, 2023). There are diverse mental health supports available to adolescents online, including websites, social media content, forums, apps, text‐based counselling, and AI‐based chatbots (Pretorius, Chambers, Cowan, & Coyle, 2019). Not only does this content vary in format and purpose, but it also varies in quality; some online support is evidence‐based and produced by reputable organisations (e.g., charity websites, evidence‐based apps), whereas others are more informal and user‐generated (e.g., social media, forums). These latter supports may incur risk of harms for example, through malicious or triggering content (Pretorius, Chambers, Cowan, & Coyle, 2019). Nevertheless, adolescents report many benefits to engaging with online mental health support including anonymity, privacy, accessibility, and an increased sense of autonomy and control (Pretorius et al., 2019a, 2019b). Studies have started to consider how adolescents' engagement with online support affects engagement with specialist healthcare services, for example, how social media can improve communication with doctors (Naslund et al., 2016, 2019). However, none have considered this in relation to school‐based support.

Developing a more comprehensive understanding of how school‐based and online support intersect in relation to adolescent mental health is not only important from a practical perspective, but also from a theoretical perspective. Socioecological models conceptualise adolescent mental health difficulties as the product of multiple complex interactions between the adolescent and multiple inter‐related environmental systems (Bronfenbrenner, 1979; Navarro & Tudge, 2023). Within this approach, most research has focussed on *microsystemic* influences on mental health and examined how immediate and direct environmental interactions shape outcomes in positive and negative ways. Most studies have focussed on adolescents' interactions within environments independently (e.g., Pretorius, Chambers, Cowan, & Coyle, 2019, Pretorius, Chambers, Cowan, & Coyle, 2019), meaning their combined and intersecting influence—that is, the *mesosystemic* influence ‐ is under‐studied. Yet school environments have potential to influence adolescents' engagement with online environments, and vice versa. For example, school‐based media literacy interventions have been shown to mitigate against risks of online harm (Lee & Hancock, 2023; Wade et al., 2017). As such, understanding mesosystemic interactions between school‐based and online support may provide the key to improved mental health support for adolescents.

### The present study: Aims and research questions

Using a co‐produced exploratory qualitative methodology, the present study aims to examine the intersection between adolescents' engagement with school‐based and online sources of mental health support by examining both adolescents' and stakeholders' perspectives. It answers the following questions:

RQ_1._ How and why adolescents engage with mental health information and support both at school and online?
RQ_2_. What are adolescents' and adult stakeholders' perceptions of the interaction between school‐based and online mental health provision, including the risks and opportunities?

## METHOD

### Co‐production

To enable co‐production throughout the study, a youth advisory board (YAB) was established. Youth advisory board members were recruited through Kooth Digital Health Youth Leadership Team and University of York student body (*N* = 4; Age *Range* = 18–22; Gender *Girl/woman* = 2, *Boy/man* = 2; Ethnicity *White British* = 3, *Prefer not to say* = 1; Parental education^1^ *Compulsory only* = 1, *Higher secondary* = 1, *Lower tertiary* = 1, *Prefer not to say* = 1). All had attended secondary school in England and had experience engaging with online support for a variety of mental health difficulties (Anxiety = 1; Depression or low mood = 2; Eating problems = 1; Loneliness = 3; Self‐harm = 1; Stress = 3; Suicidal feelings = 1).

Funding and the broad parameters of the research (e.g., aims, qualitative approach, sample size) were already determined before the YAB were established. Once formed, the YAB were positioned as both research partners and experts through experience, and as such, were empowered to draw on their lived experience of mental health difficulties to shape research processes (Prior et al., 2022). They participated in two workshops pre‐data collection and two workshops post. In pre‐data collection workshops, the research aims and questions were presented, along with the proposed methods, for refinement. They participated in proposed focus group activities and provided input on how these may be experienced by young people (e.g., potential ethical issues) and improved. Study materials, including recruitment adverts and information sheets/consent forms were also presented, discussed and refined. In post data collection workshops, early analyses (e.g., initial coding templates and example quotes) were presented for discussion and interpretation, priorities within the dataset were identified, and implications were considered. Workshops took place via Zoom and lasted 80–103 min. Table 1 provides an overview of how workshops informed methods and analysis.

**TABLE 1:** Summary of consultation workshop discussions with youth advisory board (YAB), including recommended actions and implementation.

Summary of discussion, recommendations and actions
**Choice of participation** ‐ Recommended that participants should have choice over mode of participation (interview vs. focus group) to ensure comfort and diversify participants (Workshop 1). This choice was provided to all adolescent participants.
**Terminology** ‐ Recommended use of term “mental health difficulties” over alternates, for example, distress, problems (Workshop 1). Term used consistently on all study documents, for example, recruitment flyers, ethics documents, dissemination outputs.
**Recruitment e‐flyer** ‐ Feedback provided on range of e‐flyer formatting options and suggested text (Workshop 1). ‐ Final design and wording for recruitment flyers created to reflect pooled feedback and approved in subsequent meeting (Workshop 2)
**Ethics** ‐ Suggested changes in wording to information sheet and consent forms (Workshop 2). ‐ Recommended creating a visually‐appealing youth‐friendly flyer summarising study and key ethical considerations (Workshop 2). Flyer approved via email. ‐ Feedback provided on safety plans (i.e., how distress in interviews and focus groups would be managed) and integrated into final version of safety plan (Workshop 2) ‐ Assessed the suitability of support organisations offered to (Workshop 2).
**Design of methods** ‐ Advised not all participants will engage with photo‐elicitation activity (Workshop 1). Back‐up activity created by research team based on suggestions and approved by YAB (Workshop 2). ‐ Prompts for persona creation refined (Workshop 1). ‐ Ideation activity unanimously felt to be too difficult. Collaboratively discussed how to improve this, resulting in a shorter scaffolded version of the activity being used in the study (Workshop 2).
**Analysis** ‐ Feedback provided on initial coding template created by the research team following a presentation of codes and example quotes for example, coding quotes around identifying with celebrity mental health struggles in podcasts as examples of a need to connect rather than credibility (Workshop 3). ‐ Refined coding template to incorporate suggestions from YAB
**Dissemination** ‐ Feedback provided on key findings from analysis to identify those that should be prioritised in project outputs (Workshop 4). ‐ Co‐production of findings briefs for teachers focussing on how to improve school‐based mental health support and education (Workshop 4). Final resources approved by email.

*Note*: Materials described in this table can be accessed on the Open Science Framework (https://osf.io/cjrsd/) including the recruitment flyer, information sheet, summary and consent forms, interview and focus group schedule and findings briefs for teachers.

### Participants

#### Adolescents

Twenty seven adolescents (Age *M* = 15.42, *SD* = 1.58, *Range* = 12–18) participated in the study. Our aim was to recruit a maximally diverse sample of participants within the funding timeframe, including from diverse state schools across England (*n* = 19; see Table 3) and social media (*n* = 8). All self‐reported that they had experienced mental health difficulties and had engaged in online help‐seeking while in school (see Table 2 for more detail on sample including mental health experiences and demographic characteristics).

**TABLE 2:** Adolescent participant information.

	*N*	%
**Gender**		
‐ Boy	4	15
‐ Girl	19	70
‐ Non‐binary	2	7
‐ Prefer not to say	1	4
**Ethnicity**		
‐ White British	22	81
‐ White & Asian	2	7
‐ Other (Romanian White)	1	4
‐ Prefer not to say	1	4
**Mental health difficulties experienced**		
‐ Anxiety	20	74
‐ Stress	17	63
‐ Depressive symptoms/low mood	14	52
‐ Loneliness	12	44
‐ Body image	12	44
‐ Eating problems	9	33
‐ Anger	8	30
‐ Suicidal feelings	8	30
‐ Self‐harm	4	15
‐ Substance misuse	3	11
‐ Family issues	1	4
‐ Prefer not to say	1	4
**School support accessed**		
‐ Friends	17	63
‐ School staff (not teacher, nurse or counsellor)	15	56
‐ Teacher	11	41
‐ School nurse/counsellor	10	37
‐ Someone who works with the school	6	22
‐ Mental health lessons	4	15
‐ School assembly	2	7
‐ Information display	2	7
**Online support accessed**		
‐ Messaging app (e.g., Whatsapp)	20	74
‐ Social media		
‐ Youtube	16	59
‐ Instagram	16	59
‐ TikTok	11	41
‐ Snapchat	9	33
‐ Pinterest	8	30
‐ Facebook	5	19
‐ Twitter	4	15
‐ Tumblr	1	4
‐ Websites	8	30
‐ Online counselling	4	15
‐ Forums/Reddit	4	15
‐ Apps	3	11
‐ Blogs	1	4

**TABLE 3:** Details of participating schools.

	School 1	School 2	School 3	School 4	School 5
Participants (*n)*	3	7	7	1	1
Proportion of students experiencing disadvantage^a^	Higher than national average	Higher than national average	Higher than national average	Lower than national average	N/A
Geographic location	Urban, North‐East England	Rural, South‐West England	Urban, North‐West England	Urban, North‐East England	Urban, North‐East England
School size	700	3000	1200	1100	3000
School gender composition	Mixed‐gender	Mixed‐gender	Mixed‐gender	Mixed‐gender	Mixed‐gender

^a^ Based on proportion of students receiving pupil premium, which is given to children with low family income, children of military personnel and looked after children/children adopted from care.

#### Stakeholders

Fourteen stakeholders were recruited through author networks, social media adverts and word‐of‐mouth to participate in one‐on‐one interviews. All stakeholders were based in England and provided mental health support to adolescents, including in education (*n* = 4), digital services for example, online counselling service providers (*n* = 3), charities (*n* = 3) and healthcare (*n* = 4). Adult stakeholders were included in the study since they are directly involved in mesosystemic interactions and so represent a unique and insightful perspective on how such interactions shape adolescents' mental health.

### Procedure

The study received ethical approval from University of York ethics review board. Following YAB guidance (Table 1), adolescents chose their mode of participation: individual interview or focus group. They provided informed consent in advance (additional parental consent was sought for those under 16) and completed a short question capturing demographic information, mental health difficulties experienced and past engagement with support. Stakeholders participated in interviews only and also provided informed consent. All were audio‐recorded then transcribed verbatim.

#### Interviews (adolescents)

Semi‐structured interviews examined adolescents' personal experiences of engaging with mental health support. Photo‐elicitation was used to prompt discussion (Leonard & McKnight, 2015); participants were instructed to bring 10 photographs to the interview that reflected their engagement with mental health support at home or at school. Adolescents then selected a photograph and were asked a series of prompts to elicit their experiences of engagement with mental health support across settings, as well as how different supports interact. Following advice from the YAB (Table 1), an alternative activity was provided for those who chose not to bring photographs. These adolescents were presented with a deck of cards with different types of mental health support written on (e.g., teachers, websites), selected one and were probed using the same series of prompts. Ten interviews were conducted via Zoom, lasting 29–49 min.

#### Focus groups (adolescents)

**FIGURE 1:**
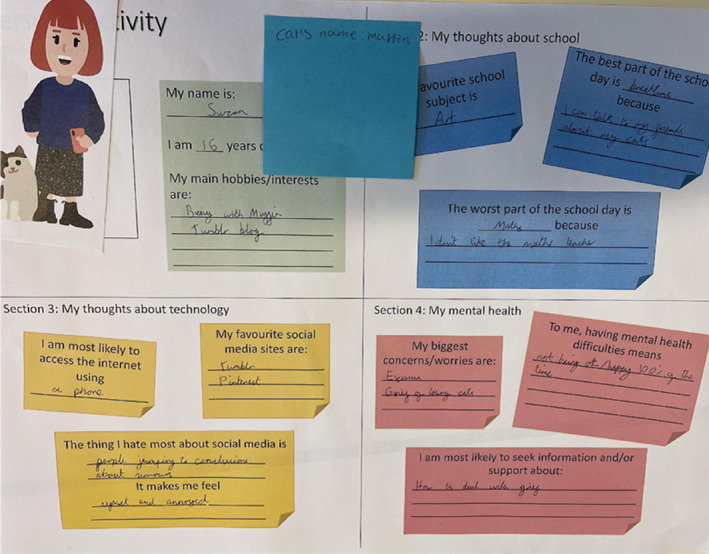
Completed persona.

Creative techniques commonly used in co‐design research, were used to engage participants in semi‐structured group discussions. First, participants collectively created a “persona” (Honary et al., 2019) to represent an adolescent of a “similar age who is experiencing mental health difficulties” by following a series of prompts (see Figure 1). Personas enable the group discussion of sensitive topics while allowing for dissociation from personal experiences (Honary et al., 2019, 2020), thus facilitating participant comfort and were used across activities. Next, participants engaged in experience‐mapping (Honary et al., 2020) where they mapped when, where and how individuals “like their persona” might engage with mental health support in a typical school day, considering online and school‐based support. Prompts encouraged adolescents to reflect on how different types of support might ‘intersect’ (i.e., influence, replace, combine) with one another. Third, participants engaged in evaluations of different sources of mental health support by (a) ranking them according to usefulness, trustworthiness and accessibility, and (b) engaging in a more detailed evaluation of a subset of supports. Evaluation tasks fostered critical comparisons between types of support, including how/why they may be preferred. Last, participants engaged in modified version of an ideation activity (Kitson et al., 2023), where they worked as a group to ideate five different ways in which school‐based and online services could work, and then critically appraised these ideas, including in terms of risks and opportunities. Five focus groups were conducted (*n* = three to four p/group), lasting 65–79 min.

#### Interviews (stakeholders)

Stakeholders participated in semi‐structured interviews either on Zoom or in schools. The flexible interview schedule included questions on their perceptions of adolescents' engagement with school‐based and online support, as well as the intersection between them, with a particular focus on strengths, weaknesses, risks and opportunities. Fourteen interviews were conducted, lasting 24–61 min.

### Analytic procedure

Data was analysed using template analysis through a critical realist lens (Brooks et al., 2015). First, Author 1 and 2 developed an initial coding template; a priori themes were developed based on the research questions and initial reflections during data collection, and then refined by coding a subset of data. The initial coding template and indicative quotes were then presented to the YAB for feedback and revised accordingly. Author 1 then coded the remaining dataset, refining the template iteratively throughout, including by inserting, deleting, combining and/or renaming themes. Author 1 and 2 reviewed the final analysis and coding template to ensure that it accurately reflected the dataset and research questions.

To ensure robust and trustworthy research processes, we adhered to Thomas and Magilvy (2011)'s four principles of qualitative research rigour. Specifically, we ensure [1] *Credibility* by working closely with the YAB on study design and findings interpretation (see Table 1), [2] *Transferability* through detailed description of participants and schools (see Tables 2 and 3), [3] *Dependability* through transparent reporting including by making study materials available on the Open Science Framework (see https://osf.io/cjrsd/?view_only=88c66daf1f3e4ba987397c2a13b8975f), [4] *Confirmability* by integrating independent scrutiny of the analysis by Author two into the analysis process to check that it accurately reflects dataset (e.g., checking how extracts were coded, drafts against the data). Examples of changes made following this scrutiny include highlighting the added value of virtual support for adolescents with communication difficulties (Theme 2) and emphasising the discrepancies in stakeholders' perceptions of adolescents as critical consumers of online information (Theme 4).

## ANALYSIS

Through template analysis, we developed four themes within the dataset, which are outlined in this section. Codes are provided alongside quotes, wherein STK = Adult Stakeholder, YPI = Young Person (Interview), YPF = Young person (Focus group). The two digit numbers after these digits are used as a proxy for participant ID.

### Support is abundant, but accessing what you need when you need it is tough

Adolescents and stakeholders described valuing the diversity of mental health support available to adolescents across context, which had potential to cater to the diversity of adolescents' mental health needs. However, both described how difficult it was for adolescents to find what they need, when they need it. Some support, especially in schools, was perceived as too generic to be useful:

They [teachers delivering compulsory mental health education] just do it about identifying mental health and what the different problems are, but they never do anything really about how you can help yourself

[YPI14].

In contrast, information online was more likely to be relevant and useful, but this was not always easy to access: “*Finding it on social media is a lot easier* […] *most people don't really know where they can go or how easy it is to find support*” [YPI29]. The abundance of support online was positioned as overwhelming: “*There's a lot of content that exist, a lot of organisations* […] *for some young people it could just be really overwhelming knowing where to start*” [STK16]. As such, adolescents described how finding useful information was often coincidence: “*You see, I kind of accidentally followed this person* [on TikTok] *who is actually quite useful, she does online therapy*” [YPF33]. Both described how more detailed discussion of the online mental health space, including how to access support, was needed in schools:

There’s a lot of lessons on how to be safe online and what to avoid but, nobody really speaks about the benefits of social media and being able to google things [related to mental health] people don’t speak enough about just how easy it is to access help in all different environments […] speaking more about the benefits as well as how to be safe would be helpful

[YP09].

Specialist support, especially for more complex or severe difficulties, was positioned as especially difficult to access across contexts: “*we're trying to support students for all sorts of different reasons and there's not enough access to that next level*” [STK09]. Adolescents described experiencing long waitlists for these services; “*There's such a long wait, there's so many people who need help, and obviously one person* [school counsellor] *can only do so much* […] *getting the help you need can take a long time*” [YPI29]. Stakeholders expressed concerns about how schools in particular were facing increased demands with limited resources: “*More and more is getting put on schools. And schools aren't getting any more time* […] *or money*” [STK19]. Though online services could alleviate some pressure, they weren't always suitable: “*Things like medical help and medication and treatment which goes beyond just talking ‐ that's hard to find online and it's something that people can get quite wrong just by looking online”* [YPI29].

There were other barriers to accessing the right support at the right time. School‐based support was only available during school hours, so online support was needed outside of this: “*You might be able to access that at random times of the day when you're struggling or it's just more convenient generally*” [YPI16]. Furthermore, adolescents struggled to motivate themselves to access support when they needed it: “*When you're that burned out slab, it's kind of hard to think about that sort of thing*” [YPF12].

### Human connection is vital and can be achieved in diverse ways

Most adolescents described how their desire for human connection motivated their engagement with support online and in‐school. They wanted to share their feelings with others; feel listened to and understood:

let's say I was speaking to her [favourite teacher] about an argument that I had, she just listens because it's good to just talk to someone even if you're not getting any advice back because she just listens to it.

[YPI33].

This sense of connection was typically achieved by verbal interaction online or in‐school. Other times, it was achieved through text‐based interactions (e.g., text‐based counselling): “*Knowing that you're talking to someone and they're listening to you and they're seeing your responses and things, I think that definitely helps people*.” [YPI29]. Connection could also be achieved through non‐interactive support. Adolescents described how reading or listening to the personal stories of other people with experiences mental health difficulties (e.g., through podcasts) could also make them feel less alone and normalise their experiences:

YPF23: *if they look up to the person* [talking about their mental health in a podcast], *they’re going to feel better if they’ve experienced it too*

YPF25: *Because of the relatedness of the experience* […]

YPF23: *Yeah, it’s becoming more common to talk about it, rather than be judged by it.* […]

YPF22: *Well, it’s first‐hand experiences of people ‐ you can relate to it more.*

Adolescents and stakeholders described circumstances where engaging with school‐based support could be preferable to online support in achieving this sense of connection, and vice versa. School‐based connections were more useful when adolescents were experiencing severe difficulties: “*if you're looking for anything more serious, I think you should go to someone that you trust first than online, because if you do online, then you're keeping it to yourself and it could make it worse*.” [YPI08]. In contrast, virtual connections were advantageous for overcoming geographic boundaries: “*in everyday life you don't always find people who have the same problems as you but it's good to have that connection with someone who is feeling similar to you, so you feel like you're not alone*.” [YPI29]. Virtual support was also described as less daunting; “s*ome people might get a bit nervous speaking in person*” [YPI33], especially for adolescents with communication difficulties: “*A lot of young people, especially autistic children, are terrified of picking up the phone*” [STK15]. Individual differences among adolescents also influenced how they sought connection:

Everyone’s different because some people want someone to talk to ‐want that physical. Some people don't want to talk to anybody [at school] but are quite happy to sit on a computer and talk.

[YPI24].

Some people are fine not doing that [talking to someone] and have the motivation to do it independently, but some people value the relationship a lot more. [STK18].

Connection could also be achieved through peer support. Peer support was available both in school and online and was perceived as easy to access; “*one of the reasons why we have to listen to our friends so much* [is] *because we're not gonna get any information from anyone else anywhere quicker because it's just so slow*” [YPI39]. Peer support was often reciprocal; adolescents with mental health difficulties described both giving and receiving support: “*I have a couple of friends who would vent to me about whatever they think and then I can just help them out and give them a little bit of advice and then send them on their way*” [YPI25].

### Striving for autonomy and control

Adolescents described striving for autonomy and control when engaging with support. Both adolescents and stakeholders perceived value in adolescents' ability to choose which support to engage with. Being able to self‐refer to services was essential to adolescents' sense of autonomy, especially where parents were unsupportive:

In our parents' generation, there is still a lot of stigma, and I think that sometimes that barrier stops a lot of people from getting help, because their own mental health struggles aren’t recognised by their parent‐ if they’re able to refer themselves without having parents involved is really important, because I think that’s one of the hardest conversations to have.

[YPI06]

Despite wanting to initiate support on their terms, adolescents didn't always find this easy, especially in schools: “*It takes quite a lot of bravery to go and say, ‘I need this help, please could you help me?’*” [YPI29]. Digital channels, especially text‐based services, were seen as preferable first steps and seeking support online often provided adolescents with a gateway to school support: “*we try to work with them digitally to help get them support face to face as well*” [STK26]. Many schools recognised this preference and provided online text‐based self‐referral for adolescents, which were positively received:

At my school […] it’s a ‘tell us what's on your mind’ thing […] and it's really great. I haven't used it before, but I know a couple of my friends have, and it's basically a little forum but you just put your name and whatever you want to talk about and then someone will see it and then help you with it […] I think that's really, really great

[YPI25].

Stakeholders and adolescents both described how support for adolescents should be both discreet and confidential. Adolescents described wanting control over who knew about their difficulties; “*I don't want to tell them* [school staff] *that I have anxiety and then them go off and make a big deal and tell everybody* […] *that's not what I want. I just want someone to know*” [YPI14]. This was a challenge, especially at school. First, safeguarding responsibilities were a concern; adolescents were worried that disclosures made in school would automatically be passed onto parents or authorities:

My form tutor’s always saying ‘if I hear something of concern I have to report it’ […] I don’t really know what’s classed as concern because if I was talking about like how I felt and stuff I don’t know if that would be classed as concerned and then I’d be reported.

[YPI14]

Second, mental health stigma from peers was perceived as a problem in school: “People find it hard to say, ‘Oh I'm just going to talk to the mental health nurse’ […] they don't want to be judged by their friend group or be pushed away.” [YPI29]. Stigma was worsened by the high visibility of mental health help‐seeking in schools, and was perceived as a barrier to accessing support, especially for boys: “When it comes to male mental health, they want to appear strong in front of their friends‐they want to appear powerful, and I think children seeing them then going to access support‐to a teacher‐it can seem a bit weak.” [STK26].

In contrast, discreet and confidential support was easier to access online. This was largely due to the anonymity afforded by most online support: “*It's consistently what young people tell us is the main reason why they use* [ANONYMOUS] *is the anonymity*” [STK03]. Adolescents' desire for anonymity often determined their choice of support; sometimes they wanted to be anonymous for example, the start of their help‐seeking journey or for less severe mental health problems. In other circumstances, they valued being known; “*But obviously, that* [anonymous support] *might not help in the long run if they need special helpful treatment. So maybe at the start very anonymous but then by the end to have the option to not be*” [YPI25].

### Navigating credibility and trust across contexts

Adolescents and stakeholders described how adolescents navigated issues related to quality of support across settings, including trust and credibility. Both online and in‐school, adolescents described they were more likely to engage with support from individuals (e.g., teachers, peers) whom they were already knew and trusted. However, adolescents still questioned their knowledge and credentials in relation to mental health, and were sceptical of support they received:

We've [my peer group] all come to the understanding that we all have the best of intentions but we don't always actually know what we’re talking about so take everything with a grain of salt

[YPI39].

I don’t necessarily think they [subject teacher] could give you the best advice, because that’s not really what they’re trained in, they’re just trained in to teach that subject

[YPI04].

Adolescents described being similarly sceptical of online support. They recognised that people online may not be who they say they are; “*Even if the person on the forum was a doctor, I wouldn't know. Therefore, I might not trust them*” [YPI07], and turned to other cues as indicators of trustworthiness, such as perceived site regulation; “[Youtube] *is more trustworthy than TikTok because* […] *if someone spread some really bad misinformation, 10 other YouTubers, really big ones, would be like, “Hey, this is the dumbest thing ever. Don't do this* […]” *Whereas TikTok that doesn't happen*.” [YPI39]. They also described how engaging with mental health support online could be harmful, due to the lack of regulation and algorithms that dictated social media feeds and search engines:

YPF12: It's a bit of a dice roll sometimes. Half the time you'll get to an actual therapist who's made a bunch of videos and the other half you'll get somebody trying to sell you on the new Scientology […]

YPF13: I think that's how Andrew Tate got so big.

YPF14: Well yeah, he was you know self‐help ‐ but taken to a very weird place because people who need self‐help are often mentally in that frail state.

Thus, adolescents perceived themselves as critical consumers of online information. Some stakeholders shared this view. However, others disagreed: “*Young people are just very trusting in nature and naïve because they probably haven't experienced the world in the depth that it needs to be experienced yet*” [STK16]. Irrespective of their position, most stakeholders perceived supporting adolescents to navigate different types of mental health support was a crucial part of their role: “*it's about us having really clear go‐to places so you don't go to places and get the wrong messaging, which is obviously what we control less of when it's in the digital sphere*” [STK06]. However, these efforts were not always effective “*they show stuff on these TV screens* [at school…] *they're just peripheral stuff, so you don't interact with it*” [YP06].

## DISCUSSION

Adopting a co‐produced exploratory qualitative methodology, the present study examined adolescents' and stakeholders' perceptions of how and why adolescents use different types of school‐based and online mental health support [RQ1] and also the mesosystemic interaction between these two microsystems [RQ2]. Findings highlight how despite having a diverse range of mental health support available across settings, this support was not always relevant or accessible (Theme 1). Nevertheless, the diversity of support meant that some of adolescents' needs, including the need for connection (Theme 2), were more likely to be met. The desire for autonomy and control, including over disclosure (Theme 3), as well as considerations about trust and credibility (Theme 4), influenced adolescents' choice of support. Across themes, adolescents and stakeholders highlighted the potential for more collaborative mesosystemic interactions between schools and online support.

Findings show how adolescents' need for mental health support can be met in different ways by different types of support. Consistent with past research (e.g., Pretorius, Chambers, & Coyle, 2019), Pretorius, Chambers, & Coyle, 2019), dolescents' need for connection was a key motivation underpinning their engagement with mental health services. The extent to which different types of support met this need was dependent on the affordances of the support and the characteristics of the adolescent (e.g., readiness/ability to talk, severity of symptoms). For example, adolescents who preferred to engage with support anonymously could fulfil their need for connection through text‐based counselling services or listening to mental health podcasts, whereas others who were more comfortable verbalising their difficulties might find their needs better served by a favourite teacher in school. Thus, adolescents and stakeholders valued the diversity of mental health support available to adolescents. Such variety may be especially important when meeting the needs of neurodivergent youth.

Adolescents also valued the ability to choose which supports they engaged with. Crucially, no single form of support was described as sufficient, and instead adolescents engaged with multiple types of support in self‐directed and complementary ways, drawing on their respective strengths and weaknesses. This description is consistent with accounts of engagement with other types of health support across settings (Bell et al., 2024). However, adolescents were only able to benefit from this diversity of mental support if they were aware of what support was on offer and possessed the skills and confidence to access it. Efforts to enhance adolescents' awareness of mental health support and how to access them were described (e.g., signposting), but these were not always effective.

Engagement with online support did affect engagement with school support. Sometimes online support was used when school support was not appropriate, for example, when wanting to remain anonymous. Other times, online support was used as a gateway to school support for example, using text‐based online services to build confidence prior to engaging with schools. Alternatively, online support was used to augment school‐support, for example, providing out‐of‐hours care. In all of these instances, adolescents use of online support was perceived positively as complementing school support rather than replacing it, as theorised by Burns et al. (2016). This is in contrast to past research, which have construed engagement with online support over face‐to‐face services negatively, for example, by delaying access to specialist services (Gowen, 2013). Text‐based online communication plays a particularly important role and warrants further investigation.

Challenges to engagement with mental health support were identified across settings. At school there were concerns surrounding disclosures and visibility of engagement with services, consistent with past research on stigma (Bowers et al., 2013). In online settings, concerns were more around harmful content that could worsen mental health (e.g., graphic content) and disinformation (e.g., harmful ideological messages disguised as mental health support), consistent with emerging research (Starvaggi et al., 2024). Improved interaction between schools and online mental health support could help overcome these issues. For example, mental health awareness campaigns on social media could be developed and evaluated to see how they impact on stigma in schools.

Some problems persisted across settings. Most notably, adolescents and stakeholder described the lack of specialist support available to adolescents. These perceptions reflect a reality; specialist mental health services in the UK have faced repeated funding cuts (Docherty & Thornicroft, 2015) and suggest that the most vulnerable adolescents (i.e., those with more complex, severe or established difficulties) may be the least likely to access the support they need. While online services could work more responsively with schools to develop specialist support, it is clear that government policy needs to prioritise youth mental health and reverse funding cuts.

### Methodological implications

By collaborating with adolescents on the design and analysis of the study, voices of adolescents with lived experience of mental health difficulties were incorporated into the research process (Mayer & McKenzie, 2017). Crucially, recommendations from the YAB improved how adolescent participants *experienced* the research process and ensured that youth voice was represented in the interpretation of findings. Though we co‐produced recruitment strategies with the mixed‐gender YAB and made efforts to diversify the sample by recruiting from both urban and rural schools across England, the final sample was small and non‐diverse, comprising mainly White girls. This may be due to the framing of study; to take part, adolescents needed to have accessed mental health support and studies show that racially minoritized groups and boys are less likely to do this (Allouche et al., 2021; Wang et al., 2020). Thus, the benefits of coproduction are not without limits and the transferability of findings are limited. More research that examines how other adolescents (e.g., boys, racially minoritized youth) perceive mesosystemic interactions between schools and online mental health services is needed.

Our use of creative techniques to prompt discussion in focus groups also have important methodological implications. The techniques used are typically used in co‐design research to examine perceptions of technology and/or services (e.g., Honary et al., 2019, 2020). In addition, they create a more democratic and collaborative environment for young and vulnerable research participants, with less researcher involvement (Hodson et al., 2023). In the present study, we further demonstrate their utility in answering research questions pertinent to youth mental health.

### Practical implications

While the practical implications of our findings are limited by the exploratory nature of the present study, they point to several potentially beneficial directions for future work in this space. Specifically, schools could explore the benefit of an enhanced mental health curriculum that encompasses the online environment. Based on our findings, such curriculum could involve increased critical literacy of online mental health information and support, practical lessons that guide adolescents on how to access mental health support and discussions around implications of anonymity. Furthermore, online services could explore the added value of engaging more with schools in the delivery mental health education and the creation of opportunities for mental health education beyond the classroom. Indeed, some promising examples of digital mental health literacy tools already exist (Curran et al., 2023) and their potential for implementation in school contexts should be considered. Engaging young people in the co‐production of these education efforts to ensure authenticity and acceptability is recommended.

## AUTHOR CONTRIBUTIONS

**Beth Teresa Bell**: Conceptualization; Data curation; Formal analysis; Funding acquisition; Investigation; Methodology; Project administration; Writing ‐ original draft; Writing ‐ review & editing. **Laura Fox**: Data curation; Formal analysis; Investigation; Methodology; Writing ‐ review & editing. **Louisa Salhi**: Funding acquisition; Investigation; Writing ‐ review & editing. **Daniel Fitton**: Conceptualization; Funding acquisition; Methodology; Writing ‐ review & editing.

## CONFLICT OF INTEREST STATEMENT

The authors declare no conflicts of interest.

## ETHICAL CONSIDERATIONS

Ethics approval was granted by the Education Ethics Committee (Ref FC22/3). All participants provided informed consent. Additional parental consent was obtained for participants under the age of 16.

## Data Availability

Data, where permission for sharing was provided participants, is available on the Open Science Framework (https://osf.io/cjrsd/), along with other study materials including interview and focus group schedules, ethics forms, codebooks and dissemination materials.

## ENDNOTE

^1^
Highest parental education level used here as an indicator of socioeconomic status.
